# Ingroup Favoritism Surrounding COVID-19 Vaccinations in the Hispanic Communities: Experimental Study

**DOI:** 10.2196/71188

**Published:** 2025-05-27

**Authors:** Juwon Hwang, Asya Cooley, Skye Cooley, Robert Hinck

**Affiliations:** 1 Department of Mass Communication, Advertising and Public Relations College of Communication Boston University Boston, MA United States; 2 Core Faculty Center on Emerging Infectious Diseases, Boston University Boston, MA United States; 3 School of Media and Strategic Communications Oklahoma State University Stillwater United States; 4 Leadership and Innovation Institute Air University Montgomery United States

**Keywords:** COVID-19 vaccinations, Hispanics, ingroup favoritism, social identity theory, Spanish

## Abstract

**Background:**

Hispanic communities have been disproportionately affected by the COVID-19 pandemic. In addition to elevated health risks and burdens, these populations have faced persistent barriers to accessing accurate, timely information regarding the pandemic’s trajectory, including vaccine-related updates. To address these challenges, it is crucial to examine the conditions under which Hispanics are most likely to seek information about COVID-19 vaccinations.

**Objective:**

Grounded in social identity theory and self-categorization theory, the primary goal of this study is to investigate how ethnic and linguistic cues influence information-seeking preferences related to COVID-19 vaccinations among Hispanic individuals. The first aim is to compare Hispanic and non-Hispanic participants in terms of their preferences for COVID-19 vaccine-related social media pages, in which the ethnicity of individuals shown in the images (Hispanic vs non-Hispanic) and the language in the text (Spanish vs English) vary. The second aim is to identify which combination of ethnic imagery and language in the text is most preferred among Hispanic participants when seeking COVID-19 vaccination information.

**Methods:**

A total of 936 participants (Hispanic: n=448; non-Hispanic: n=488) were included in the study. We created experimental social media group pages modeled after Facebook groups, in which the ethnicity of individuals shown in the imagery and the language used in the text were manipulated. A total of 4 conditions were developed: (1) Hispanic imagery with Spanish text, (2) non-Hispanic imagery with Spanish text, (3) Hispanic imagery with English text, and (4) non-Hispanic imagery with English text. Participants were asked to indicate the extent to which they would be willing to seek help from each social media group page, under the assumption that they were looking for information or assistance related to the COVID-19 vaccine, regardless of their actual vaccination status. A between-subjects ANOVA and a one-way repeated-measures ANOVA were conducted to analyze the data.

**Results:**

The findings indicated that Hispanic participants significantly preferred social media pages featuring Hispanic imagery and Spanish text compared to non-Hispanic participants. Moreover, a page with non-Hispanic imagery and English text was less preferred by Hispanic than by non-Hispanic individuals. Among Hispanic participants, the condition featuring Hispanic imagery and Spanish text emerged as the most favored, particularly when compared to conditions featuring non-Hispanic imagery paired with either Spanish or English text. Notably, there was no significant difference between the preference for the condition with Hispanic imagery and Spanish text and the condition with Hispanic imagery and English text, suggesting that imagery may have a stronger influence than language in shaping preferences.

**Conclusions:**

These results suggest that incorporating ethnic and language cues that reflect the target audience’s identity can enhance the effectiveness of public health messaging, particularly in efforts to improve information engagement among Hispanic populations.

## Introduction

### Background

The COVID-19 pandemic has brought new attention to long-standing inequities faced by racial and ethnic minority populations [1]. Hispanic communities, along with other underserved and socioeconomically disadvantaged groups, have been disproportionately affected by the COVID-19 pandemic [2-5]. Researchers specifically identify coexisting medical conditions, limited access to health care, and language barriers as contributing factors [6]. In addition to experiencing a higher risk and burden associated with the virus, Hispanic communities have encountered persistent difficulties in accessing accurate and timely information regarding the pandemic’s progression, including updates about vaccines and effective mitigation measures [1]. For instance, during the early days of the pandemic, public information websites, hotlines, and computer-based systems for testing and vaccination appointments were primarily offered in English [1]. Furthermore, the historical lack of trust in health care and government institutions, stemming from institutional racism and harm [7-10], has further complicated efforts to disseminate essential information such as COVID-19 vaccination updates. To address these challenges, researchers and practitioners should understand Hispanic communities’ preferences for receiving COVID-19 vaccination information with attention to cultural and linguistic cues.

The phenomenon of ingroup favoritism [11] presents a promising area of research that could enhance our understanding of these preferences. According to both social identity theory [12] and self-categorization theory [13], individuals typically gravitate toward a group with which they identify [11]. An important assumption of this is that the presence of an outgroup is necessary for ingroup categorization and the subsequent emergence of ingroup favoritism [13]. A body of literature supports the ingroup favoritism phenomenon [11]. In most of these studies, group membership is either experimentally manipulated (eg, [14,15]) or defined by existing factors, such as gender [16-18] and race and ethnicity [19,20].

Unlike previous research focusing on racial differences using White and Black participants [19], studies examining ethnic differences (ie, Hispanic vs non-Hispanic) require caution, as Hispanic individuals may identify with diverse racial backgrounds, such as White, Black, Indigenous, and multiracial identities [21]. Although racial differences within the Hispanic population can significantly influence factors such as health outcomes [22], these differences may be less central in the context of ingroup favoritism. Ingroup identification among Hispanic individuals often occurs in contrast to non-Hispanic outgroups, with shared cultural or ethnic markers serving as primary indicators of group membership [23]. For example, while Hispanic individuals may vary in racial identity (eg, White, Black, and Indigenous), a White Hispanic individual may still perceive a darker-skinned Hispanic as part of their ingroup if shared ethnic or cultural features are recognizable. Thus, racial heterogeneity within the Hispanic population may not significantly disrupt perceptions of ingroup affiliation, as shared cultural identity can override racial distinctions when evaluating group membership in intergroup comparison contexts [24].

Therefore, this study uses a computer-based experimental design modeled after Facebook group pages, in which we manipulate the ethnicity of individuals shown in the images (Hispanic vs non-Hispanic) and the language used in the text (Spanish vs English). First, we examine whether preferences for social media pages featuring Hispanic imagery and Spanish text differ based on participants’ self-identified ethnicity. We also compare the opposite combination—whether preferences for pages featuring non-Hispanic imagery and English text vary by participants’ ethnicity. Next, we explore which specific combination of ethnic imagery and language is most preferred among Hispanic participants when seeking COVID-19–related information. By exploring these research questions, this study aims to offer insights into the communication strategies and message conditions under which COVID-19 vaccination information can be most effectively delivered to Hispanic communities.

### Ingroup Favoritism, Social Identity Theory, and Self-Categorization Theory

Individuals’ self-perceptions are often based on and influenced by their group identity perceptions [12]. Groups are a pervasive feature of our social lives [11]. People tend to interact with others who share common group identities, such as nations, races, religions, and political parties [11]. Furthermore, individuals evaluate ingroup members more positively than outgroup members [25,26], reward ingroup members more than outgroup members [12], and work harder to accomplish ingroup goals [27]. Such positive bias toward one’s ingroup is termed ingroup favoritism [11,19].

A total of 2 theoretical perspectives have directed research on intergroup favoritism: social identity theory [12,28] and self-categorization theory ([13]; for summary, see also [29]). The self-categorization theory posits that categorizing oneself and others into distinct social categories is sufficient to elicit ingroup favoritism [11,30]. Similarly, social identity theory distinguishes between personal identity, which is how individuals think and feel about their unique characteristics, and social identity, which is part of an individual’s self-concept derived from their knowledge of the membership of a social group [11,31]. Social identity theory further suggests that individuals self-categorize as belonging to a specific group in the presence of an outgroup. Since individuals are motivated to maintain a positive social identity, simple categorization triggers thoughts, feelings, and behaviors that seek to positively differentiate the ingroup from the outgroup [11,12].

An important premise in social identity theory is the metacontrast principle [13], which states that ingroup favoritism requires the presence of an outgroup and intergroup comparison [11]. According to this theory, ingroup categorization and subsequent favoritism do not occur without the presence of an outgroup [11,32]. Only in the presence of a salient outgroup, positive social identity striving should manifest itself in tendencies to cooperate with ingroup members more than with outgroup members [33,34].

Recent studies during and after the COVID-19 pandemic suggest that health crises may amplify ingroup identification. For instance, individuals are more likely to perceive health messaging as credible when the messenger shares their racial or ethnic identity, especially during high-stress periods like the pandemic [35]. Evidence also indicates that culturally tailored narratives, such as those reflecting a Hispanic cultural perspective and delivered in Spanish, can significantly enhance vaccine confidence among Hispanic individuals, further underscoring the role of identity alignment in effective communication between public health agencies and the public during the pandemic [36].

While research on intergroup favoritism has been well established, there remain significant gaps in the literature. Racial and ethnic comparisons need further attention since Hispanic individuals may identify with various races. Prior research has inadequately tested intergroup bias in the context of racial and ethnic differences, failing to consider ethnic identification. For example, all White participants were used to assess White and Hispanic characters in negative media content [37]. In another study, both Hispanic and White participants evaluated positive and negative news stories featuring only Hispanic characters [38]. Consequently, several scholars urge the need for more research on the relationships between Hispanic and non-Hispanic populations [39,40].

In addition, one of the most critical factors characterizing Hispanic individuals’ ingroup identities is the use of Spanish, as culturally and linguistically relevant messaging serves as a conduit to amplify ingroup favoritism. Research indicates that racial and ethnic minorities tend to trust information more when presented in their native language, whereas universal or population-wide messages can rapidly become outdated and are generally less effective in reaching diverse communities [1]. Effective public health messaging—such as that used by the Centers for Disease Control and Prevention (CDC) during the pandemic—can significantly influence positive vaccine attitudes when it is perceived as trustworthy and culturally attuned [41]. Recent studies have reinforced this pattern within the context of COVID-19. For instance, Spanish-language public health messages about vaccines were not only more trusted but also more likely to influence behavior among Hispanic populations [42]. Similarly, linguistic congruence in health campaigns enhances perceived empathy, trust, and ingroup connection [43]. However, despite this growing body of evidence, few studies have directly examined how language differences affect ingroup favoritism—especially when comparing responses to ingroup versus outgroup communicators.

Moreover, crisis conditions can foster a social-psychological environment that amplifies ingroup favoritism, as uncertainty and fear often increase individuals’ reliance on familiar group identities. According to social identity theory, people under threat or stress are more likely to draw psychological support from salient group memberships, using them as a buffer against anxiety and insecurity [44]. Empirical studies support this notion: during events such as natural disasters, terrorist attacks, and pandemics, individuals tend to exhibit stronger ingroup affiliation, greater trust in fellow ingroup members, and a preference for ingroup-aligned information and leadership [45,46]. For instance, a recent COVID-19 study—though not focused on vaccination—found that individuals were more likely to comply with health guidelines when those messages came from ingroup members or culturally relevant figures [47].

Grounded in the concept of ingroup favoritism guided by social identity theory and self-categorization theory, the first aim of this study is to examine whether Hispanic participants, compared to non-Hispanic counterparts, show a stronger preference for seeking COVID-19 vaccine information from social media pages featuring Hispanic visuals and Spanish text. Conversely, we also assess whether Hispanic participants exhibit a lower preference for pages that include non-Hispanic visuals and English text. The second aim is to examine which combination of visual ethnicity and language—(1) Hispanic imagery with Spanish text, (2) Hispanic imagery with English text, (3) non-Hispanic imagery with Spanish text, or (4) non-Hispanic imagery with English text—is most preferred by Hispanic participants. Accordingly, we propose the following 2 research questions (RQ).

RQ1: How do Hispanic and non-Hispanic participants differ in their preferences for social media pages communicating COVID-19 vaccine information, based on the ethnicity portrayed in the images (Hispanic vs non-Hispanic) and the language used in the text (Spanish vs English)?

RQ2: Among Hispanic participants, which combination of imagery (Hispanic vs non-Hispanic) and language (Spanish vs English) on social media pages is most preferred when seeking COVID-19–related information?

## Methods

### Data Collection and Recruitment

We conducted a within-subjects computer-based survey experiment, recruiting Hispanic and non-Hispanic participants via Amazon Mechanical Turk (MTurk). MTurk, a crowdsourcing platform that connects researchers with participants who complete tasks for compensation, often yields data quality comparable to that of traditional research methods, especially when appropriate vetting and cleaning procedures are applied [48]. In addition, MTurk provides more demographically diverse samples than traditional student-based samples [49,50].

To recruit participants, we developed 2 targeted outreach strategies on MTurk: one aimed at Hispanic individuals and the other at non-Hispanic individuals. Hispanic participants were recruited via Spanish-language advertisements and were eligible only if they self-identified as Hispanic. The survey was translated into Spanish and pretested to ensure clarity and reliability for participants with limited English proficiency [51,52]. Non-Hispanic participants were recruited through English-language advertisements and self-identified Hispanic individuals were excluded. A total of 2 independent recruitment samples were initially obtained (519 Hispanic participants and 574 non-Hispanic participants) prior to applying exclusion criteria. The non-Hispanic sample was collected over 2 days (January 24-25, 2022), whereas collecting a comparable Hispanic sample required 7 days (February 1-8, 2022). This extended recruitment period for Hispanic participants aligns with prior research noting the increased difficulty of recruiting ethnically diverse samples on MTurk [53].

All participants were required to be adults residing in the United States. For the Hispanic sample, Spanish fluency was required. For the non-Hispanic group, there were no language restrictions. To ensure data quality, we only included participants with an MTurk human intelligence task (HIT) approval rate of 98% or higher—a widely used threshold for ensuring participant reliability. Participants were excluded if they failed attention checks, did not complete the survey, had a HIT approval rate below 98%, identified as Hispanic in the non-Hispanic group, or failed to identify as Hispanic in the Hispanic group—resulting in a final sample of 448 Hispanic and 488 non-Hispanic participants.

### Participants: Hispanic Sample

Of the 519 Hispanic respondents who participated in the experimental survey, 71 were excluded for not completing the entire survey, yielding a final sample of 448 participants. The average age of Hispanic participants was 34.10 (10.32) years. Additional demographic details are presented in Table 1.

**Table 1:** Hispanic participant demographic data (N=448).

Characteristics		Participants
Age (years), mean (SD)		34.10 (10.32)
**Gender, n (%)**		
	Female	230 (51.3)
	Male	215 (48)
**Race and ethnicity, n (%)**		
	White/Caucasian (non-Hispanic)	0 (0)
	Hispanic	448 (100)
	Black or African American	0 (0)
	Asian	0 (0)
	Native American	0 (0)
	Other	0 (0)
**Education, n (%)**		
	Less than high school	19 (4.2)
	Graduate high school or equivalent	37 (8.3)
	Some college, no degree	57 (12.7)
	Associate degree	92 (20.5)
	Bachelor’s degree	243 (54.2)
**Family income, n (%)**		
	Less than US $29,999	38 (8.5)
	US $30,000-US $39,999	58 (12.9)
	US $40,000-US $49,999	64 (14.3)
	US $50,000-US $59,999	86 (19.2)
	US $60,000-US $69,999	47 (10.5)
	US $70,000-US $79,999	91 (20.3)
	US $80,000 or more	64 (14.3)

### Participants: Non-Hispanic Sample

Of the 574 individuals who initially participated in the experiment, 25 were excluded for identifying as Hispanic and 61 for not completing the entire survey. This resulted in a final sample of 488 non-Hispanic participants. The sample included 421 White, 54 Black, 9 Asian, and 13 Native American participants, with a mean age of 38.57 (SD 11.48) years. Additional demographic details are presented in Table 2.

**Table 2:** Non-Hispanic participant demographic data (N=488).

Characteristics			Participants
Age (years), mean (SD)			38.57 (11.48)
**Gender, n (%)**			
	Female	210 (43)	
	Male	278 (57)	
**Race and ethnicity, n (%)**			
	White (non-Hispanic)	421 (86.3)	
	Hispanic	0 (0)	
	Black or African American	54 (11.1)	
	Asian	9 (1.8)	
	Native American	13 (2.7)	
	Other	4 (0.8)	
**Education, n (%)**			
	Less than high school	3 (0.5)	
	Graduate high school or equivalent	20 (3.6)	
	**S**ome college, no degree	31 (5.6)	
	Associate degree	20 (3.6)	
	Bachelor’s degree	323 (58.8)	
**Family income, n (%)**			
	Less than US $29,999	45 (8.2)	
	US $30,000-US $39,999	73 (13.3)	
	US $40,000-US $49,999	81 (14.8)	
	US $50,000-US $59,999	96 (17.5)	
	US $60,000-US $69,999	52 (9.5)	
	US $70,000-US $79,999	74 (13.5)	
	US $80,000 or more	67 (12.2)	

### Experimental Social Media Group Pages

Experimental social media group pages modeled after real “Facebook COVID-19 vaccination help groups” were developed to enhance realism. These mockups were not hosted on an actual social media platform. Instead, they were displayed as static images embedded within the computer-based survey. Group names were drawn directly from existing Facebook groups. While images and language on the front pages were systematically manipulated, other elements—such as member count (3.7k) and group type (public)—were held constant to isolate the effects of the experimental conditions. To minimize order effects inherent in a within-subjects design, the order in which Facebook group pages were presented was randomized for each participant.

### Measures

Participants were asked to view 6 curated Facebook group pages offering assistance with scheduling the COVID-19 vaccine. They were instructed to imagine that they needed help related to the COVID-19 vaccine, regardless of their actual vaccination status. For each page, participants rated their willingness to seek help using a 7-point scale in response to the prompt: “Please rate your preference where you would like to get some help related to the COVID-19 vaccine for each group” (1=strongly not prefer to 7=strongly prefer).

### Analytic Strategy

This study uses a computer-based experimental design to compare participants’ preferences for social media pages that vary by the ethnicity depicted in images (Hispanic vs non-Hispanic) and the language used (Spanish vs English), based on their self-reported ethnicity (Hispanic vs non-Hispanic).

To examine RQ1, an ANOVA was conducted with participant ethnicity (Hispanic vs non-Hispanic) as a between-subjects factor. Preferences for social media pages featuring Hispanic individuals and Spanish text, as well as those featuring non-Hispanic individuals and English text, were analyzed separately as outcomes.

Next, to examine RQ2, a one-way repeated measures ANOVA was conducted to assess Hispanic participants’ preferences for social media pages featuring (1) Hispanic individuals with Spanish text, (2) Hispanic individuals with English text, (3) non-Hispanic individuals with Spanish text, and (4) non-Hispanic individuals with English text. Analyses were conducted using SPSS (version 27; IBM Corp).

### Ethical Considerations

The study was approved as exempt by the Oklahoma State University institutional review board (IRB-21-429). Informed consent was obtained from all participants. Data were anonymized to ensure privacy and confidentiality. Participants received a small monetary compensation.

## Results

For RQ1, the findings from ANOVA showed a significant impact of the between-group factor (*F*_1935_=39.87, *P*<.001, η^2^=.041), indicating Hispanic participants preferred social media pages featuring Hispanics in the images and Spanish text (mean 5.52, SD 1.28) more than did non-Hispanic participants (mean 4.91, SD 1.67; Table 3). To further examine RQ1, we conducted another ANOVA with participant ethnicity (Hispanic vs non-Hispanic) as a between-group factor and the preference for the social media pages featuring non-Hispanic individuals in the image and English text as an outcome. A significant impact of the between-group factor also emerged (*F*_1935_=7.29, *P*=.004, η^2^=.008) indicating Hispanic participants had a lower preference for the social media pages featuring non-Hispanic individuals in the image and English text (mean 5.22, SD 1.27) than did non-Hispanic individuals (mean 5.43, SD 1.06; Table 3). Taken together, these findings suggest that Hispanic and non-Hispanic participants differ significantly in their preferences for the visual and linguistic cues presented on social media pages communicating COVID-19 vaccine information. Specifically, Hispanic participants were more inclined to prefer pages that featured co-ethnic imagery and native language, and less inclined to prefer pages featuring non-Hispanic imagery and English text, relative to their non-Hispanic counterparts.

**Table 3:** Comparison of social media preferences for COVID-19–related information between Hispanic and non-Hispanic participants.

Condition	Hispanic (N=448), mean (SD)	Non-Hispanic (N=488), mean (SD)
Hispanic imagery with Spanish text	5.52 (1.28)	4.91 (1.67)
Hispanic imagery with English text	5.51 (1.10)	5.56 (1.02)
Non-Hispanic imagery with Spanish text	5.29 (1.46)	4.87 (1.73)
Non-Hispanic imagery with English text	5.22 (1.27)	5.43 (1.06)

Next, to examine RQ2, the results from a one-way repeated measures ANOVA provided evidence to reject the null hypothesis (Wilks Lambda=.93; *F*_3445_=11.43, *P*<.001, η^2^=.07). Follow-up comparisons revealed that 3 pairwise differences were significant. Specifically, Hispanic participants showed a stronger preference for social media pages featuring Hispanic individuals in the image and Spanish text (mean 5.52, SD 1.28) compared to those featuring non-Hispanic individuals in the image and Spanish text (mean 5.29, SD 1.46; *P*=.004) and non-Hispanic individuals in the image and English text (mean 5.22, SD 1.27; *P*<.001). However, there was no statistically significant difference in preference between pages featuring Hispanic individuals in the image with Spanish text (mean 5.52, SD 1.28) and those with Hispanic individuals in the image with English text (mean 5.51, SD 1.10; *P*=.90), suggesting that the ethnicity depicted in the image may be more influential than language in shaping participants’ preferences (Table 3). Likewise, Hispanic participants expressed significantly lower preference for pages featuring non-Hispanic individuals in the image and English text (mean 5.22, SD 1.27) compared to those featuring Hispanic individuals in the image and Spanish text (mean 5.52, SD 1.28; *P*<.001) or English text (mean 5.51, SD 1.10; *P*<.001), but not compared to those featuring non-Hispanic individuals in the image and Spanish text (mean 5.29, SD 1.46; *P*=.29). These findings reinforce the notion that visual representation (image ethnicity) may play a more critical role than language in determining user preferences for vaccine-related social media content (Table 3).

## Discussion

### Principal Findings

This experimental study, grounded in ingroup favoritism theory, examined Hispanic preferences for receiving COVID-19 vaccination information [13]. Specifically, it compared the preferences of Hispanic and non-Hispanic participants for social media pages that varied in the ethnicity of individuals depicted in the images (Hispanic vs non-Hispanic) and the language used in the text (Spanish vs English). The study also explored which combinations of image ethnicity and language were most preferred by Hispanic participants.

The findings are summarized as follows. Hispanic participants exhibited a significantly stronger preference for social media pages featuring Hispanic images and Spanish text compared to non-Hispanic participants, and a lower preference for pages featuring non-Hispanic images and English text. Next, Hispanic participants showed a stronger preference for pages featuring Hispanic individuals in the image and Spanish text compared to those featuring non-Hispanic individuals in the image paired with either Spanish or English text. However, no statistically significant difference emerged between preferences for pages with Hispanic individuals in the image paired with Spanish versus English text.

### Theoretical Implications

These findings offer several important theoretical contributions. First, the results provide clear evidence of ingroup favoritism among Hispanic participants, as they preferred content that aligned with their ethnic identity and native language more strongly than non-Hispanic participants. This preference for Hispanic imagery and Spanish text among Hispanic participants supports the work of previous studies on ingroup favoritism [12], which highlights how individuals are more likely to engage with content that reflects their ethnic identity [23]. Importantly, this study reinforces the findings of previous research by illustrating that ingroup favoritism is applied to the context of promoting health-related behaviors, such as COVID-19 vaccination, within historically underserved communities. This suggests that incorporating ethnic identity into public health messaging is essential for fostering trust and engagement in marginalized populations.

The results suggest that both ethnic identity and language cues play important roles in shaping message preferences, as evidenced by the strongest preference for content featuring Hispanic imagery with Spanish text. At the same time, ethnic identity appears to be the more dominant factor, given that no significant difference was found between Spanish and English text when the image featured someone from the Hispanic participants’ ethnic group. This aligns with prior research indicating that ethnic imagery serves as a salient and enduring marker of identity, often more immediately recognizable than language [54].

### Practical Implications for Designing Public Health Messages for Ethnic Minority Communities

These findings provide practical insights into effectively tailoring health communications to resonate with Hispanic communities. First, organizations aiming to engage Hispanic audiences, whether in public health, education, or commercial settings, should prioritize visual ethnic representation in their materials. Including imagery that reflects the ethnic identity of the target audience can significantly enhance message relevance and engagement. Second, the findings highlight that language cues are most effective when paired with ethnically congruent visuals. Hispanic participants preferred messages combining Spanish text with Hispanic imagery, suggesting that language alone may not be sufficient to foster trust or connection. To enhance engagement, communicators should integrate both cultural and linguistic cues in their messaging.

### Limitations and Future Directions

One limitation of this study is the lack of subgroup analysis within the non-Hispanic sample. Although comparisons were made between Hispanic and non-Hispanic participants, the latter group included racially diverse individuals (eg, Black and Asian) whose responses were not analyzed separately. However, this limitation mainly affects the non-Hispanic group; the Hispanic group’s responses still provide a valid assessment of ingroup preferences. In addition, our design did not fully capture the linguistic diversity within US Hispanic communities, as no formal assessment of English proficiency or the extent of bilingualism was conducted. While a high level of English proficiency among Hispanic respondents recruited through MTurk is assumed, given that most US-based MTurk workers are fluent in English [55,56], fluency levels in both English and Spanish may still vary widely, potentially influencing responses. Future research should account for these variations in bilingual proficiency when interpreting language-related effects. Third, although the presentation order of the Facebook group pages was randomized to minimize order effects in the within-subjects design, participants’ responses may still have been influenced by the sequence in which the pages appeared, potentially introducing bias. Also, the temporal imbalance in recruitment, with Hispanic participants recruited over 7 days and non-Hispanic participants over 2, may introduce cohort or environmental biases that could influence responses. Finally, the generalizability of our findings is limited by the self-selecting nature of the sample and the technological access required to participate in the MTurk survey.

### Conclusions

This study offers key insights into using ingroup favoritism to promote public health equity. Hispanic participants preferred content aligned with their ethnic and linguistic identity, emphasizing the need for culturally tailored communication. Public health efforts should reflect these preferences to effectively engage Hispanic communities.

**Acknowledgments**

This study was funded by Global Impact on behalf of the Vaccine Confidence Fund (VCF–012).

## Abbreviations

CDC: Centers for Disease Control and Prevention
HIT: human intelligence task
MTurk: Mechanical Turk
RQ: research question
