# Correction: Ingroup Favoritism Surrounding COVID-19 Vaccinations in the Hispanic Communities: Experimental Study

**DOI:** 10.2196/90652

**Published:** 2026-03-06

**Authors:** Juwon Hwang, Asya Cooley, Skye Cooley, Robert Hinck

**Affiliations:** 1 Department of Mass Communication, Advertising and Public Relations College of Communication Boston University Boston, MA United States; 2 Core Faculty Center on Emerging Infectious Diseases, Boston University Boston, MA United States; 3 School of Media and Strategic Communications Oklahoma State University Stillwater United States; 4 Leadership and Innovation Institute Air University Montgomery United States

In “Ingroup Favoritism Surrounding COVID-19 Vaccinations in the Hispanic Communities: Experimental Study” [1], the authors identified errors related to references and corresponding in-text citations.

Reference 43 was invalid and has been removed from the reference list, and the corresponding in-text citation has been removed. The affected statement is now supported by the following reference:

Daggett A, Abdollahi S, Hashemzadeh M. The effect of language concordance on health care relationship trust score. Cureus. May 26, 2023;15(5):e39530 [doi: 10.7759/cureus.39530] [Medline: 37366455]

Reference 53 was invalid and has been removed from the reference list, and the corresponding in-text citation has been removed. The affected statement is now supported by the following reference:

Chandler J, Rosenzweig C, Moss AJ, Robinson J, Litman L. Online panels in social science research: expanding sampling methods beyond Mechanical Turk. Behav Res Methods. Oct 2019;51(5):2022-2038. [doi: 10.3758/s13428-019-01273-7] [Medline: 31512174]

Reference 54 was invalid and has been removed from the reference list, and the corresponding in-text citation has been removed.

References 55 and 56 were invalid entries of the same reference and have been removed from the reference list, and the corresponding in-text citations have been removed. The affected statement is now supported by the following reference:

Khan H, Lee R, Lockshin L. Do ethnic cues improve advertising effectiveness for ethnic consumers? Australasian Marketing J. Aug 2015;23(3):218-226. [doi: 10.1016/j.ausmj.2015.07.001]

Reference 44 was incorrectly attributed and has been corrected to the following reference:

Haslam C, Jetten J, Cruwys T, Dingle G, Haslam SA. The New Psychology of Health: Unlocking the Social Cure. Routledge; 2018. [doi: 10.4324/9781315648569]

Reference 58 was incorrectly attributed and has been corrected to the following reference:

Ipeirotis P. Demographics of Mechanical Turk. New York University; Mar 2010. URL: https://ipeirotis.org/wp-content/uploads/2012/02/CeDER-10-01.pdf [Accessed 2026-02-20]

The correction will appear in the online version of the paper on the JMIR Publications website together with the publication of this correction notice. Because this was made after submission to PubMed, PubMed Central, and other full-text repositories, the corrected article has also been resubmitted to those repositories.

## References

[1] Hwang Juwon, Cooley Asya, Cooley Skye, Hinck Robert (2025). Ingroup Favoritism Surrounding COVID-19 Vaccinations in the Hispanic Communities: Experimental Study. J Med Internet Res.

